# Iodide of Potassium in Erysipelas

**Published:** 1873-10

**Authors:** A. H. Hiatt

**Affiliations:** Office, 125 Clark St., Chicago; Wheaton, Ill.


ARTICLE V.—*Iodide of Potassium in Erysipelas.* By A. H. HIATT, M.D., Wheaton, Ill.

Having used iodide of potassium in the treatment of erysipelas for the last ten years with the best results, I deem it right to lay the fruit of my experience upon this subject before the profession, and the more so, as I do not see this remedy for erysipelas recommended, or even referred to in any of the works on the practice of medicine which I have read.

The variety of the disease upon which I base my remarks particularly, and present my treatment, is the phlegmonous, though the remedy is equally efficacious in other varieties in which I have tried it.

It is when the disease makes its appearance first upon the side of the nose or the face, passing rapidly over the contiguous parts, until the entire face and scalp are invested, obliterating the features, enormously enlarging the head, the patient presenting a hideous and ghastly appearance, that the effects of this remedy are most promptly and happily seen. When called to such a case, whether the disease has passed to the full extent above named, or has but just commenced its ravages, I immediately prescribe the following:

| | | | |
|---|---|---|---|
| R. | Potassii Iodidi, | - - - - - - | dr. j. |
| | Aqua Pura, / Syrup Simplex, | - - - - - | *aa.* oz. j. |
| | Ess. Gaultheria, | - - - - - - | dr. ss. |

S. One teaspoonful every two hours, mixed with water; equal to seven and a half grains per dose.

This I continue until the violence of the disease is subdued, as indicated by abatement of the swelling, and mitigation of the tenderness of the tumid parts upon touch—and afterwards I continue the medicine in less quantity, or at longer intervals, until all traces of the disease are gone. In some very severe cases, attended with delirium, I give as much as ten grains of the iodide every two hours.

If the bowels are constipated, and the tongue has a brown coat, I administer fifteen grains hyd. cum creta, and follow it in six hours with seidlitz powder or citrate magnesia—a portion to be repeated every two hours until the bowels are moved.

For the prostration and failing strength, I give about two grains of quinine every four or five hours, or spts. vini gallici or old Bourbon whisky in tablespoonful doses every two, three or four hours, as the case may require.

I also insist upon the use of milk, animal broths, or other forms of nourishing food, from the commencement. If these cannot be taken, beef essence must be used.

As an external application to the inflamed and swollen parts,

I have found the following simple preparation the very best for its soothing effect merely, for I am convinced that no external application exerts any curative influence over the disease:

R. Plumb. Acetas, - - - - - - dr. j.

Aqua, }

Glycerine, } - - - - - *aa.* oz. j.

The parts to be kept moist with this.

In from twenty-four to forty-eight hours, under this treatment, the violence of the disease is usually subdued.

I can present a long list of cases treated as above, all terminating favorably, a complete recovery being effected in from two to four or five days in every instance—and which results I have seen under no other treatment.

I regard the external application of blisters, nitrate of silver, tincture of iodine, corrosive sublimate, and all other irritating and corrosive substances, as not only useless, but positively injurious, inflicting unnecessary and severe punishment upon the patient, and wasting the strength as well. I do not believe in specifics in medicine, and yet I regard iodide of potassium in erysipelas as approximating one as nearly certainly as quinine in intermittent fever.

If in the above article I shall succeed in directing the medical profession to the faithful trial of this remedy in this affection, I shall feel I have rendered invaluable service to my race.

OFFICE, 125 CLARK ST., CHICAGO.

